# The Effect of Classic Chest Physiotherapy on Postoperative Pain Scores and Hospital Stay in Patients Undergoing Off-Pump Coronary Artery Bypass Graft Surgery: A Randomized Clinical Trial

**DOI:** 10.22086/gmj.v0i0.838

**Published:** 2018-05-27

**Authors:** Mohammad Zolfaghari, Seyed Jalil Mirhosseini, Maryam Baghbeheshti, Brent A. Bauer

**Affiliations:** ^1^Student Research Committee, Shahid Sadoughi University of Medical Sciences, Yazd, Iran; ^2^Department of Cardiovascular Surgery, Afshar Hospital, Shahid Sadoughi University of Medical Sciences, Yazd, Iran; ^3^Complementary and Integrative Medicine, Division General Internal Medicine, Department of Medicine, Mayo Clinic, Rochester, Minnesota, USA

**Keywords:** Randomized Controlled Trial, Pain, Coronary Artery Bypass, Length of Stay

## Abstract

**Background:**

Chest physiotherapy (CPT) is a care that increases the mobilization of several structures from both muscle and subcutaneous tissue. We planned to investigate the effect of classic CPT on pain, fatigue, satisfaction, and hospital length of stay (LOS) in patients undergoing off-pump coronary artery bypass graft (CABG).

**Materials and Methods:**

This study was a randomized controlled trial that conducted on 50 patients undergoing elective off-pump CABG. The patients have been randomly divided into two groups; in the group A (n=25) patients received physiotherapy at a single session of classic CPT, 4 times during 2nd to 5th days for 15 minutes in every session, in the group B (n=25) patients had not protocol of this exercise therapy (control).

**Results:**

The average age of all participants was 62.08 ±9.08 years. Of the 50 patients, 33 (66%) was male. Classic CPT significantly decreased pain (P=0.04), hospital LOS (P=0.010) and could increase in patients’ satisfaction (P<0.001). However, it had no considerable effect on fatigue (P=0.725).

**Conclusion:**

According to our findings, classic CPT could improve postoperative care after off-pump CABG surgery

## Introduction

Coronary artery bypass graft (CABG) surgery is common cardiac surgery in the world that has both early and late surgical complications compares to other operations [1]. Sternal surgical complications following median sternotomy are one of the majorproblems after surgery. Pain, fatigue, and anxiety are common complications after surgery. All of these factors may compromise treatment and quality of life following surgery; therefore, decreasing of pain scales may improve outcomes after surgery [2-4].Pain is one of the most significant undesirable complications associating open surgeries. Due to the higher pain scores, the higher levels of opioid requirements and hospital level of stay (LOS) would be more problematic [5]. Patients with chest pain cannot breath normally, also opioid-induced respiratory depression may occur during acute care setting [6]; these two outcomes lead to more hospital LOS and lower satisfaction. Currently, pain is a challenge for anesthesiologists, and the techniques focus on improving post-operative pain management [7, 8]. Massage therapy (MT) is a technique that increases the mobilization of several structures from both muscle and subcutaneous tissue. This mobilization improves movement of lymph and venous return. MT may be used to promote muscle relaxation and to decrease pain which helps patients enhance their rehabilitation [9]. Classic chest physiotherapy (CPT) is one of the types of MT and recently there has been more evidence on the role of CPT on reducing pain [10]. Although some specialists in physiotherapy believe that there are some limiting factors in conducting physiotherapy for post-operative patients or even it may lead to high-risk complications in some cases [11, 12]. Nowadays, CPT is not applied routinely for the patients undergoing CABG; itis just recommended for patients who suffer from a lot of chest pain as a selective method. This study was designed to evaluate the efficacy of CPT with a standard protocol on pain, fatigue, satisfaction and hospital LOS in off-pump CABG surgery patients.

## Materials and Methods

### Design

**Figure-1:**
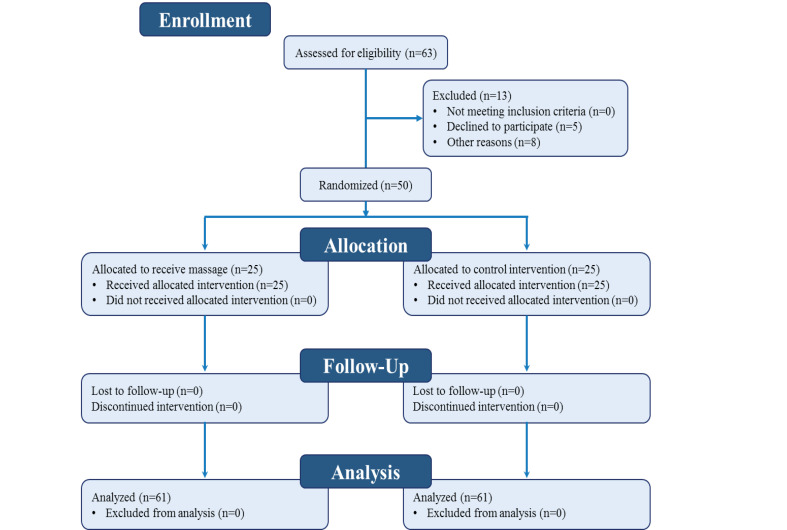
CONSORT Flow Diagram

A double-blind randomized clinical trial was conducted on 50 patients undergoing CABG. The procedure is shown in Figure-1.

### Participants

After receiving the written informed consent from patients, they participated voluntarily for off-pump CABG, at Afshar Cardiovascular Surgery Center of Yazd, Iran. Certain cardiothoracic surgeon performed all of the operations. Table-1 shows the characteristics of the participants.

**Table-1:** Demographic Information of the Participants

**Characteristics**	**Control** **(N=25)** **n(%)**	**CPT** **(N=25)** **n(%)**	**Total** **(N=50)** **n(%)**	**P-Value**
**Age ( y, Mean±SD)**	64.56±10.07	59.60±7.35	62.08±9.08	0.053
**Sex** Female Male	10 (40.0) 15 (60.0)	7 (28.0) 18 (72.0)	17 (34.0) 33 (66.0)	0.370
**HTN** Yes No	16 (64.0) 9 (36.0)	14 (56.0) 11 (44.0)	30 (60.0) 20 (40.0)	0.564
**DM** Yes No	10 (40.0) 15 (60.0)	5 (20.0) 20 (80.0)	15 (30.0) 35 (70.0)	0.123
**HLP** Yes No	10 (40.0) 15 (60.0)	14 (56.0) 11 (44.0)	24 (48.0) 26 (52.0)	0.258
**CS** Yes No	7 (28.0) 18 (72.0)	8 (32.0) 17 (68.0)	15 (30.0) 35 (70.0)	0.758
**Involved Vessels** **SVD (%)** **2VD (%)** **3VD (%)**	0 (0.0) 8 (32.0) 17 (68.0)	3 (12.0) 9 (36.0) 13 (52.0)	3 (6.0) 17 (34.0) 30 (60.0)	0.184

**CPT:**chest physiotherapy; **HTN:**hypertension; **DM:**diabetes mellitus; **HLP:**hyperlipidemia; **CS:**cigarette smoking, **SVD:**single vessel disease; **2VD:t**wo-vessel coronary artery disease; **3VD:** three-vessel coronary artery disease

### Inclusion and Exclusion Criteria

We included only those who gave written informed consent and were medically able to participate in MT postoperatively from March 5, 2016, through April 3, 2016.

Patients post off-pump CABG were similar in the inclusion criteria. Patients were excluded from the study if they met the criteria as follow: (a) history of cardiac and thoracic surgery; (b) chronic pain syndrome; (c) prolonged bleeding during hospital stay (more than 200 cc per hour); (d) psychiatric disorders; (e) history of chest trauma and deformity.

### Random Allocation

Eligible participants (n=50) were randomly allocated to the CPT or control group. The participants were divided into two groups; Patients in the group A (n=25) received physiotherapy at a single session of classic CPT 4 times during second to the fifth day for 15 minutes in every session.

In the group B (n=25) patients had not protocol of this exercise therapy (control).

To perform random allocation, a block of four was applied. Each patient in a block assigned to letters A, B, C, and D. The possible groups that could assign to CPT group were AB, AC, AD, BC, BD, and DC. Then one number from 1-6 was selected at random, for assigning to CPT group; for instance, if number 5 was selected, number 2 would be allocated to the control group.

### Blinding

The study was a double-blind trial; so, the participants were unaware of the intervention they had received. The two groups were in separate rooms to avoid possible cross-talking between them. After collecting the data, the secondary blinded researcher measured outcomes. The blinded researcher was not aware of the group of patients whether they were in the control group or CPT group.

### Measurements

The data collected using a two-part questionnaire. The first part included items about the demographic information of the participants (e.g. age, sex, cigarette smoking (CS), and the presence of hypertension (HTN), diabetes mellitus (DM), and hyperlipidemia (HLP)). Also,pre-operation data such as the number of vessels which were involved before the surgery that were single vessel disease (SVD), two-vessel (2VD), and three-vessel (3VD) coronary artery disease were recorded.

Part two included a self-report Questionnaire to evaluate the patients’ quality and degree of pain, fatigue and satisfaction.

The patients were asked to fill in a questionnaire and rate their pain, fatigue and satisfaction from 0 to 10 (0=minimum, 10=maximum). On the fifth day and before discharge, the questionnaire was filled again to determine the patients’ score finally. Pain, fatigue, and satisfaction were measured at the baseline and the end of the intervention period.

### Intervention

Patients were received follow-up care and stabled for one day in intensive care unit (ICU) after surgery. After the first postoperative day, they were transferred to the heart ward.

Thus CPT sessions must be started from the second postoperative day and only after transfer of the patients to the ward. In order to homogenize the two groups, four 15-minute sessions were determined as quiet time in days 2-5 after surgery. To preventing entering confounding factors, all of the processes of intervention was performed by a just one physiotherapist familiar with methods of physiotherapy in cardiac surgery patients. Selection of the methods of physiotherapy was on a patient-oriented basis; individualized to each patient (selected according to the psychological and physical states of the patients and other underlying conditions. Each session consisted of three parts. In the first part which lasts for 1-5 minutes,

the best conventional position wasevaluated for the patients.Indeed, every patient knows the best position according to the medical condition (such as sitting on the edge of bed, lying down with supine position, lying down with lateral decubitus position, etc.).

The position could be affected by many factors such as the ability to move, tubing and medical equipment connected to the patient and the patient’s preference. After being in the right situation, in the second part, physiotherapist tries to perform gentle and relaxant exercises to reduce anxiety and tensions of the patient. Movements of the shoulder joint (manual techniques for the shoulder) an example of the relaxant movements. This movement is beneficial after heart surgery specially in patients with a long time motionless on the bed. In the third part of each session, the physiotherapist tries to apply MT methods to help patients. These methods are according to the physiotherapist’s knowledge and opinion and due to the patient’s medical condition, the patient’s signs and symptoms as well as the patient’s tolerance. Hence, deep tissue massage, trigger point therapy, neuromuscular techniques, myofascial release, acupressure, some Swedish massage techniques, manual lymphatic drainage, and reflexology were selected. The physiotherapist would attempt not to push, pull or hit the regions which were very close or on the surgical incisions, and they avoid inflicting of physical forces on the patient’s sternum region (due to the mid-sternotomyduring operation). All techniques could be performed in other parts of the body such as the head, neck, shoulders, arms, hands, back, and legs based on the patient’s request. During the second to fourth days after surgery, to unify intervention in intervention and control groups, three 15-minute sessions entitled (silence time) for patients in the control group was considered that the patient was lying on the bed no physical activity. Also, after discharge and at follow-up period, the patients will benefit from standard medical care (such as drugs, clinical trials, and aperiodic visit by a physician), it was recommended that patients should comfort on a bed in a low-light environment, every week for thirty minutes.

### Ethical Issues

Local Medical Ethics Committee of Shahid Sadoughi University of Medical Sciences (SSU) approved the study proposal with the ID number: 746/G/T.

Moreover, this clinical trial registered in the Iranian Registry of Clinical Trials (IRCT) and allocated a unique code IRCT2016010925913N1. All of the participants signed the written informed consent.

### Data Analysis

The data were analyzed by SPSS16 software. Regarding demographic information of participants, descriptive statistics (mean, standard deviation, frequency, percentage) were calculated. The ANOVA and Chi-square tests were used for analyzed variables and to evaluate the difference between groups. A P-value< 0.05 was considered as significant.

## Results

In this study, 50 patients who underwent elective off-pump CABG were enrolled.

The average age of all participants was

62.08 ±9.08 years. In the control group, 40% (10) were female, and 60% (15) were male, and 28% (7) were female, and 72% (18) were male in the CPT group. Totally, 33 patients (66%) were male and 17 patients (34) were female. Of all the participants who were studied in this survey, 30% had DM, and 60% suffered from hypertension (HTN). In addition, 48% of them had hyperlipidemia (HLP), and 30% of them were cigarette smokers (CS). From all the participants, 6% were SVD, 34% with 2VD, and 60% with 3VD coronary artery disease (Table-1). According to the ANOVA and Chi-square results, there was a significant statistical relationship between the CPT and reducing pain (P=0.004).

On the other hand, patients who were massaged for 15 minutes complained of less pain in the 2-5 days after surgery. The mean score of pain was 1.56 in the CPT group at the final level, and it was lower in comparison with the control group (3.04).Although, CPT had no considerable effect on fatigue. The mean score of fatigue was 1.28 in the CPT group, and it was approximately close to the score in the control group (1.84).

**Figure-2:**
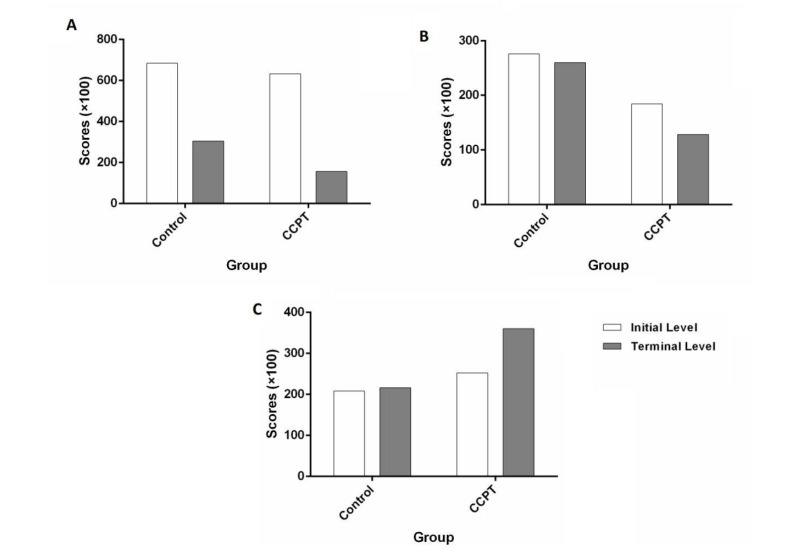
Level of pain** (A),** fatigue** (B),** and satisfaction** (C)** before and after CPT

There was no statistical relationship between the CPT and fatigue (P=0.725) (Table-2). According to the statistical outcomes, classicCPT could decrease hospital LOS; the mean score in the CPT group (6.20) was lower than the control group (7.16) (P=0.01). As mentioned above, pain and LOS make patients feel unsatisfied; in this research, pain and LOS werelower in the CPT group. Therefore, satisfaction was increased among patients in the CPT group (Figure-2).

**Table-2:** The Effect of CPT On Pain, Fatigue, and Hospital LOS in Comparison with Control Group

	**Initial Level** **(Mean±SD)**		**Terminal Level** **(Mean±SD)**		**Change in Amount** **(after-before)** **(Mean±SD)**		**P-value**
	**Ctrl**	**CPT**	**Ctrl**	**CPT**	**Ctrl**	**CPT**	
**Pain**	6.84 (1.37)	6.32 (1.74)	3.04 (1.02)	1.56 (0.76)	3.80 (1.08)	4.76 (1.16)	0.004
**Fatigue**	2.76 (0.66)	2.60 (0.70)	1.84 (0.68)	1.28 (0.67)	0.92 (0.81)	1.32 (0.98)	0.725
**LOS**	___	___	7.16 (1.40)	6.20 (1.11)	___	___	0.010
**Satisfaction**	2.08 (0.49)	2.16 (0.68)	2.52 (0.65)	3.60 (0.50)	-0.44 (0.71)	-1.44 (0.76)	<0.000

**Ctrl:** Control; **CPT:** chest physiotherapy; **LOS:** level of stay

## Discussion

The aim of this study was to deal with the effect of CPT on pain, satisfaction, fatigue and hospitalization in post-CABG patients. In this clinical trial, CPT had considerable positive effects on pain relief after CABG. Satisfaction was another important factor that could be used as an indicator of quality of life, which has been much considered in patients following major surgeries these days. At last, shorter duration of bed rest in hospital after CABG was a better way to reduce hospital costs and common complications of surgery (e.g. thromboembolism); moreover, patients often had more emotional supports and better remission when they were discharged as soon as possible.

Pain, fatigue, and anxiety are common complications after surgery. All of these factors may compromise treatment and quality of life following surgery; therefore, decreasing pain scales may improve outcomes after surgery. According to our hypothesis, performing physiotherapy on an area could increase its circulation. Therefore, there would be more oxygen provided for the tissue by increasing the red blood cell count. It also increases the immune system function in the area by providing more immune factors. These two can remove pathogenic factors and relatively heal wounds and alleviate pain [13-16].

Ucuzal M *et al*. carried out research on 70 patients undergoing breast surgery to determine the effect of foot massage on post-operative pain and found that this therapeutic method in breast surgery patients was effective in postoperative pain management [17]. Jane and Chen reflected the benefits of massage therapy on reducing pain in patients with metastatic bone and ameliorating anxiety in CHF patients, respectively [18, 19].

Also, Tarja claimed that, there was a negative relationship between massage therapy and pain in patients who were admitted in ICU [20]. Interestingly, CPT could be a multi aspect method when it is utilized in low mood. Mirmohammad sadeghi *et al*. 2013 reported better mood in 72 patients post CABG after 4 sessions of massage therapy per day. These patients were 18-75 years old [21]. Also, Streit RS studied the effectiveness of MT for the treatment of neurogenic thoracic outlet syndrome (NTOS). They used MT for treatment and found improvement in range of motion at the glenohumeral joint. Therefore, it can be useful as part of a broad approach to managing of this syndrome [22].

Castro AA *et al*. showed that CPT can minimize hospital LOS in patients hospitalized in the ICU [23]. Nowadays, physiotherapy is used to decrease hospital LOS [24, 25], pain in osteoporosis [26], chronic low back pain [27], knee osteoarthrosis [28], musculoskeletal disorders [29], open-abdominal surgery [30] and increase satisfaction. The results of this clinical trial showed a decrease in chest pain and hospital LOS and also an increase in patient satisfaction. However, since the post-operative cares after cardiac surgery are so sensitive and some of them can cause complications in healing wounds and immune system function, any intervention including physiotherapy even though having some pain-relieving effects, should be considered as stressful and with adverse effects. Physiotherapy, when performed by a specialized physiotherapist, has much fewer side-effects compared with using drugs, and it can also be a less stressful substitution. Furthermore, in this study choosing the right time for performing the physiotherapy, its repetition and selecting an appropriate method based on patient’s state, were of high importance. We tried to completely inform the patients before enrolling in the study to avoid any negative outcome resulting from their dissatisfaction. Finally, we conclude that physiotherapy can diminish chest pain and hospital LOS and increase patient’s satisfaction.

## Conclusion

Since close monitoring is necessary for patients after CABG operation, CPT could be helpful to reduce their chest pain in the department of cardiovascular surgery in Iran. However, patients often want to stay in hospital as short time as possible; therefore, postoerative CPT may partly meet their needs.

## Conflict of Interest

The authors declare that there is no conflict of interests regarding the publication of this article.
